# Supplementary material for: Ribosome profiling at isoform level reveals evolutionary conserved impacts of differential splicing on the proteome
Source: Nat Commun. 2020 Apr 14;11:1768. doi: 10.1038/s41467-020-15634-w (PMC7156646; doi:10.1038/s41467-020-15634-w)
Supplement: Supplementary file 1 — Supplementary Information [file 41467_2020_15634_MOESM1_ESM.pdf]

Supplementary Information

Ribosome profiling at isoform level reveals an evolutionary conserved impact of differential splicing on the proteome

Reixachs-Solé et al.

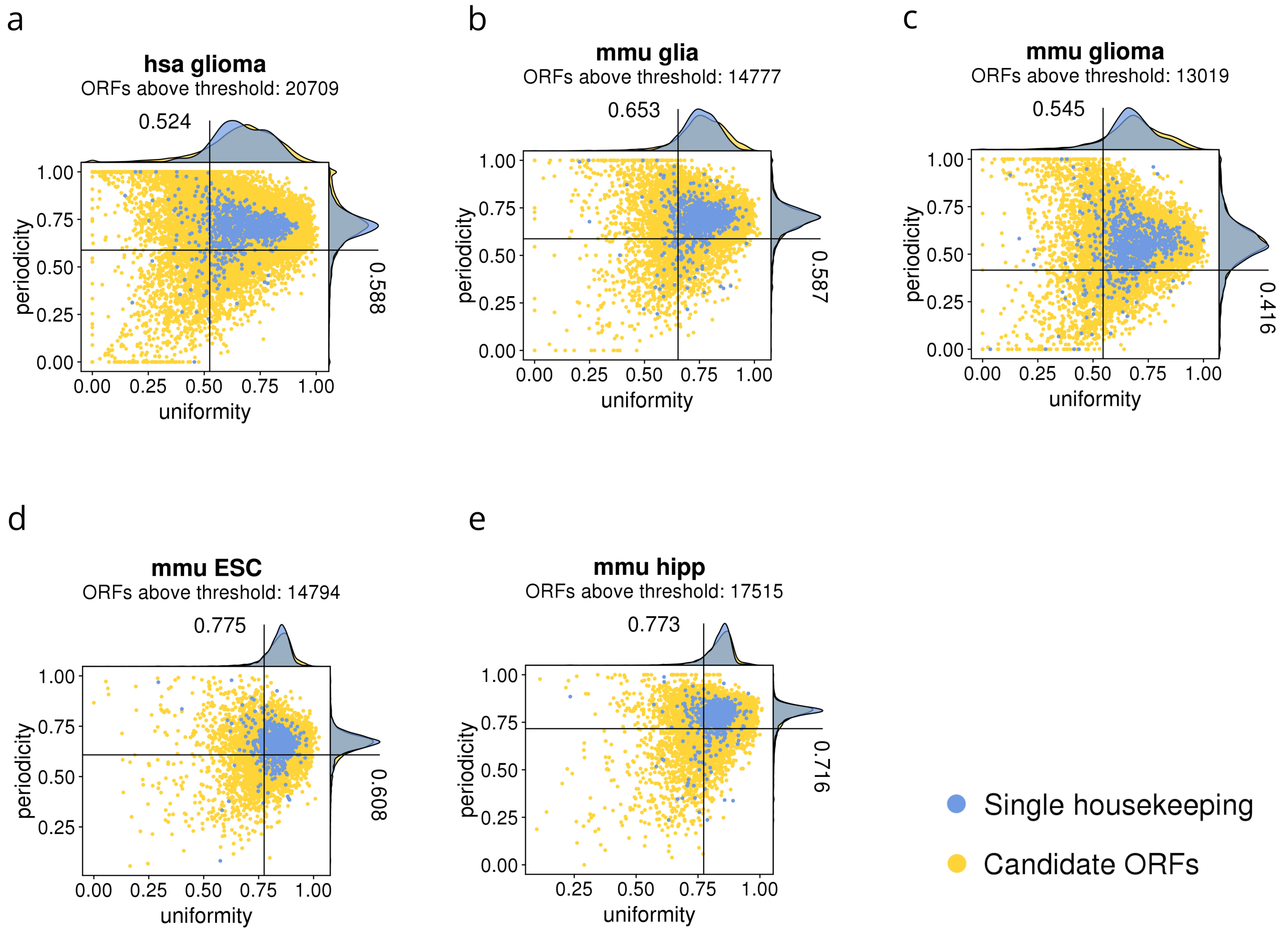

Supplementary Figure 1. Uniformity (x axis) versus periodicity (y axis) for the ORFs with with RNA expression TPM > 0.1 and at least 10 Ribo-seq reads assigned. In blue we indicate ORFs from genes defined as housekeeping singletons, and in yellow the rest of ORFs considered. Uniformity is measured as the percentage of maximum entropy and periodicity is measured in the first annotated frame. We show the data for human sample of **(a)** glioma (hsa glioma n=27294) and mouse samples of **(b)** glia (mmu glia n=18564), **(c)** glioma (mmu glioma n=16388), **(d)** Embryonic Stem Cells (mmu ESC n=19613) and **(e)** hippocampus (mmu hipp n=2203).

a

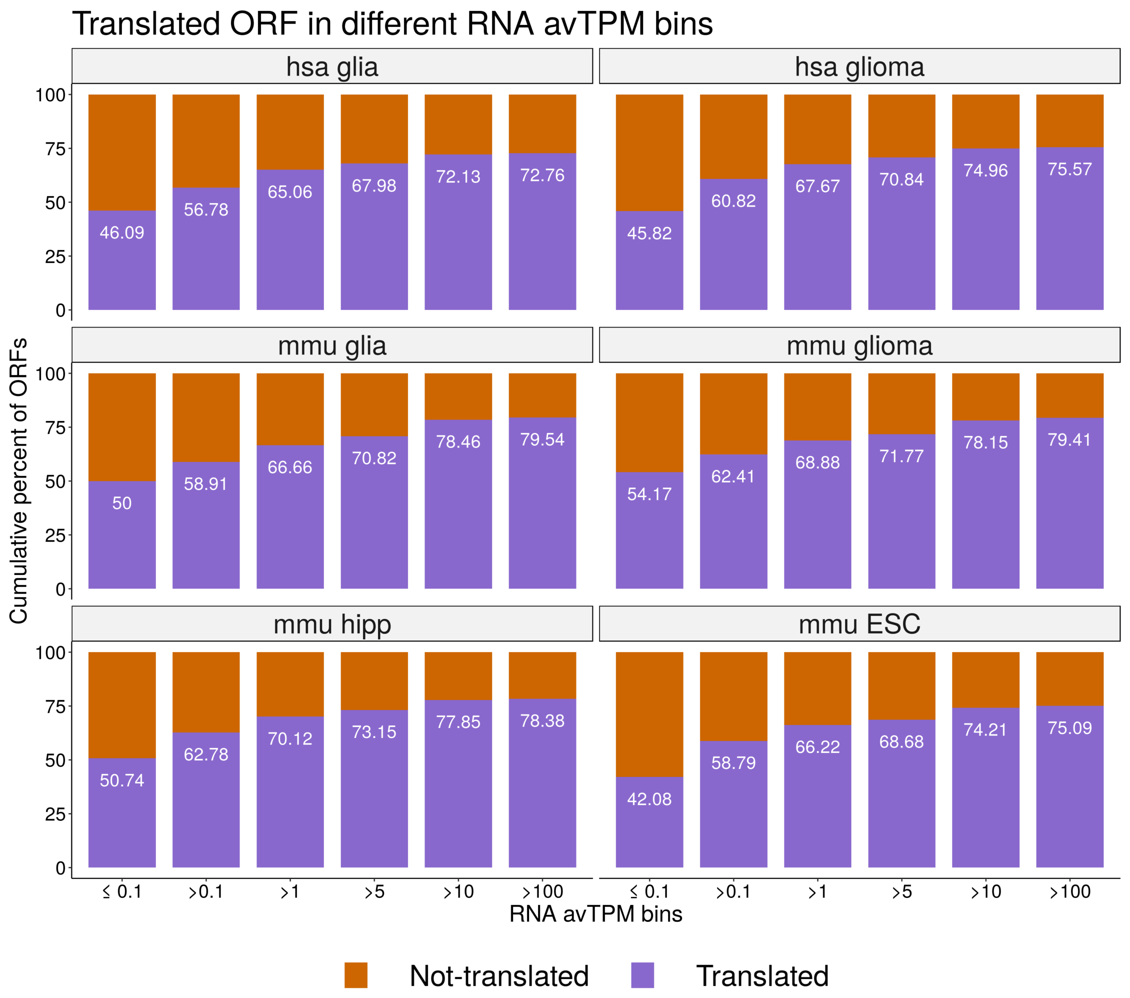

Supplementary Figure 2. (a) Cumulative percentage of ORFs above the uniformity and periodicity cut-offs established by ORQAS, therefore predicted to be translated (in purple), according to increasing cut-offs of average RNA-seq abundance values (TPM) for the human samples of glia (hsa glia) and glioma (hsas glioma) and the mouse samples of glia (mmu glia), glioma (mmu glioma), hippocampus (mmu hipp) and Embryonic Stem Cells (mmu ESC).

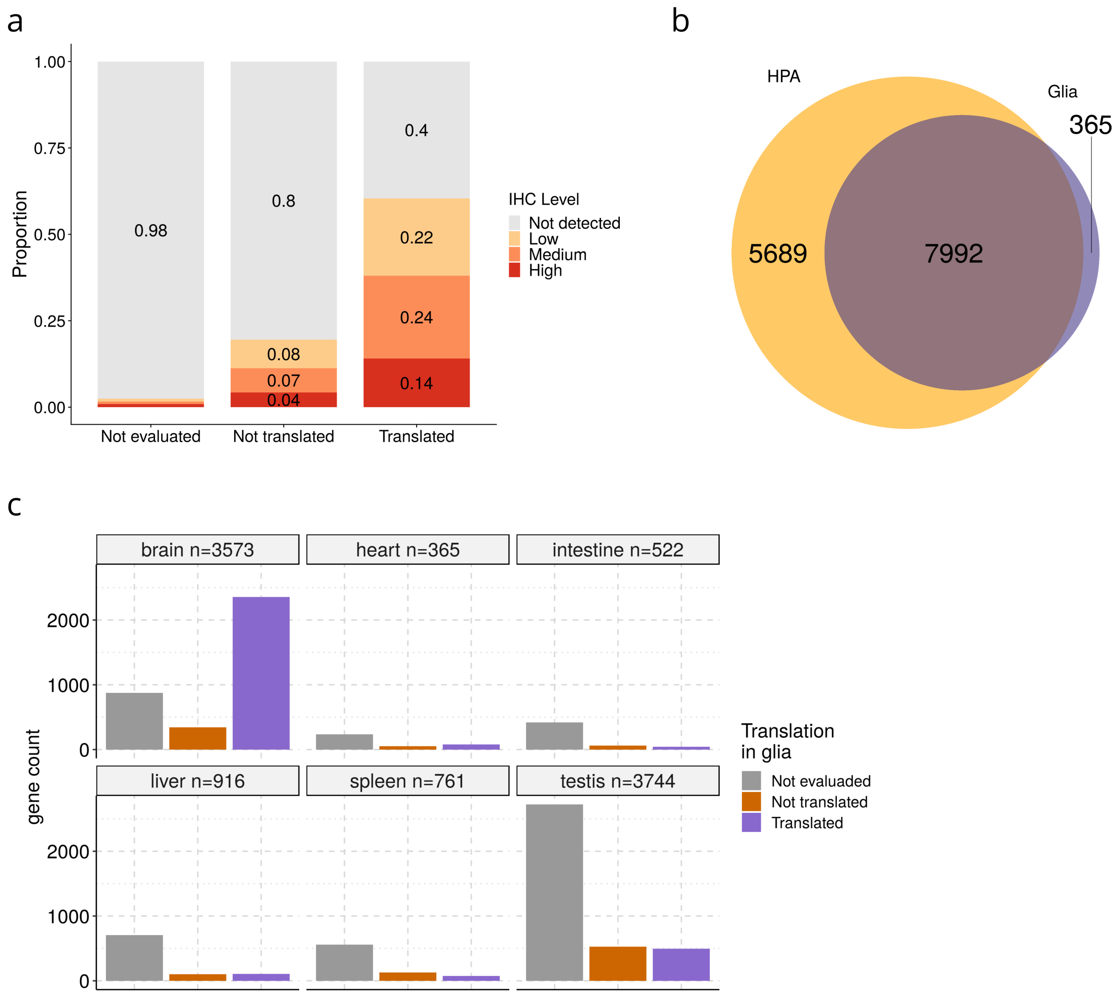

Supplementary Figure 3. (a) For the candidate translated isoforms (translated n=1942), the cases that did not pass the threshold of uniformity and periodicity (not-translated n=328), and those without enough read data to be tested (not evaluated n=564) in human glia (hsa glia), the plot shows the proportion of cases in which the corresponding gene has evidence from immunohistochemistry in cortex, separated as high, low, and medium expression, from the Human Protein Atlas. We also indicate the cases not detected in the immunohistochemistry experiments (Not detected). The comparison was made for the human glia Ribo-seq. Singletons (single-ORF genes) were not included. **(b)** The plot shows the number of genes with predicted translated ORFs that have evidence of protein expression in the Human Protein Atlas from a combination of features: Mass Spectrometry, Immunohistochemistry and Uniprot. Translation predictions correspond to human glia Ribo-seq. Singletons were not included. **(c)** For genes with tissue specific RNA and protein expression, as annotated in the Human Protein Atlas, in six different tissues (brain, heart, intestine, liver, spleen and testis), the plot shows ORQAS predictions in the human glia sample (hsa glia) for the candidate translated isoforms (translated), the cases that did not pass the threshold of uniformity and periodicity (not-translated), and those without enough read data to be tested (not evaluated).

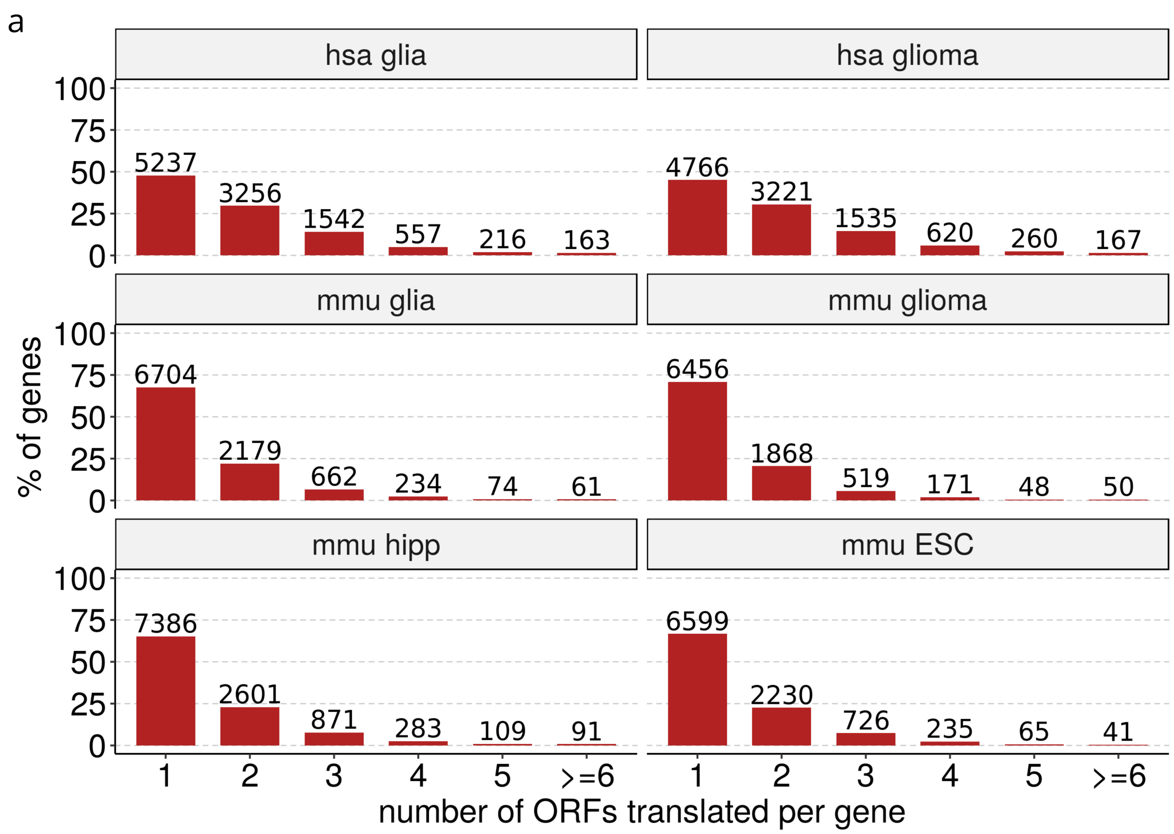

Supplementary Figure 4. (a) Distribution of the number of different ORFs translated per gene in the human samples of glia (hsa glia) and glioma (hsa glioma) and mouse samples of glia (mmu glia), glioma (mmu glioma), hippocampus (mmu hipp) and Embryonic Stem Cells (mmu ESC).

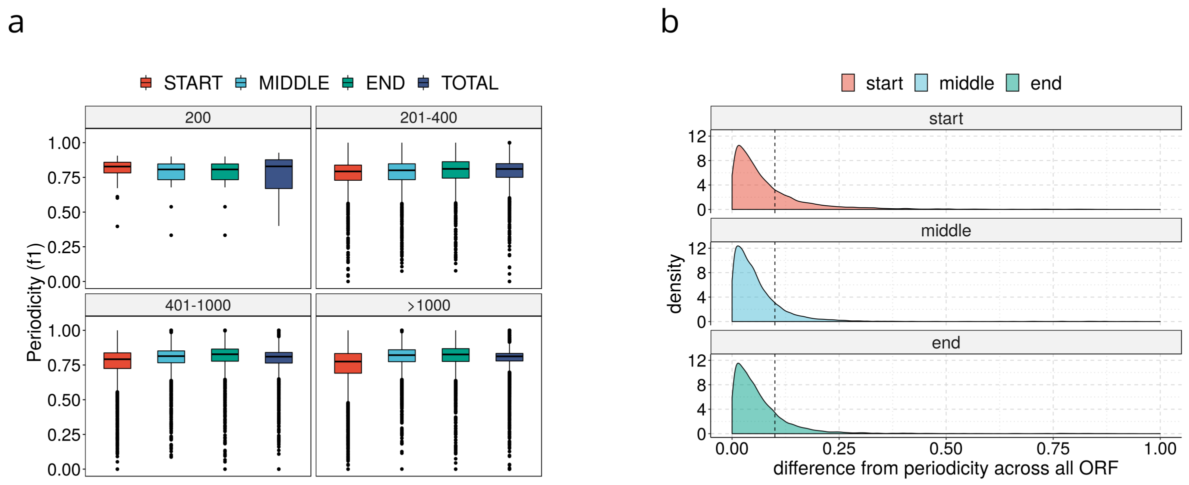

Supplementary Figure 5. (a) Distribution of periodicity values calculated for the segments resulting when partitioning the ORFs portions of equal length in the start (red), in the middle (light blue) or in the end (green) and the periodicity value calculated globally for the whole ORF length as it is used by ORQAS pipeline, grouping the ORFs according to its length (200nt, 201-400nt, 401-1000nt and >1000nt). Box boundaries correspond to the first and the third quartiles, the median is indicated by a thick black line, top and bottom whiskers extend up to 1.5 times the interquartile range to the highest and smallest values, respectively, and outliers are indicated as black dots. **(b)** Distribution of the differences of the periodicity values in each of the segments resulting when partitioning the ORFs in three portions of equal length in the start (red), in the middle (light blue) or in the end (green) and the global periodicity the whole ORF length. The dashed line is set at 0.10.

a

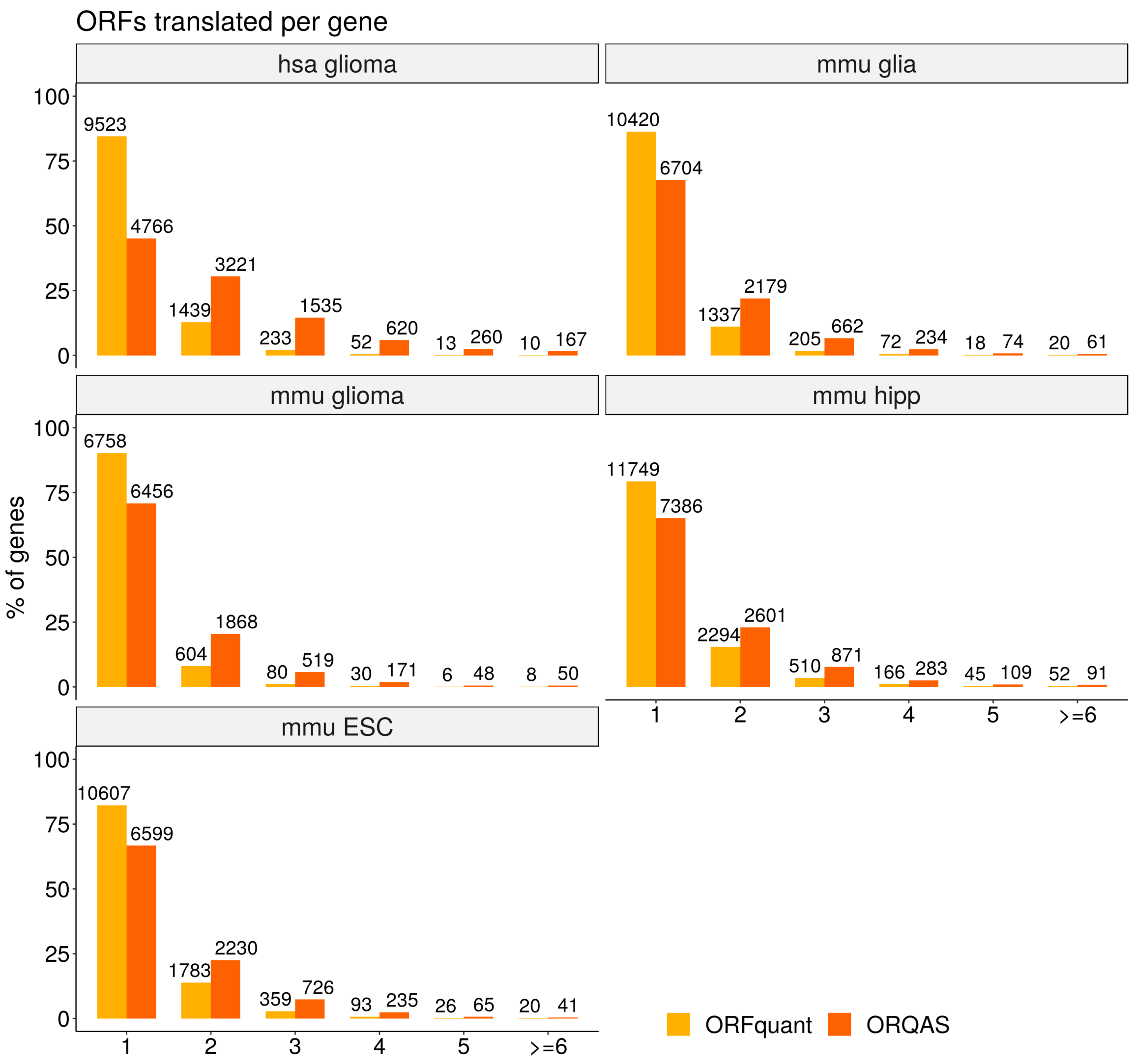

Supplementary Figure 6. (a) Distribution of the number of different ORFs translated per gene according to ORQAS (orange) and ORFquant (yellow) in the human samples of glia (hsa glia) and glioma (hsa glioma) and mouse samples of glia (mmu glia), glioma (mmu glioma), hippocampus (mmu hipp) and Embryonic Stem Cells (mmu ESC).

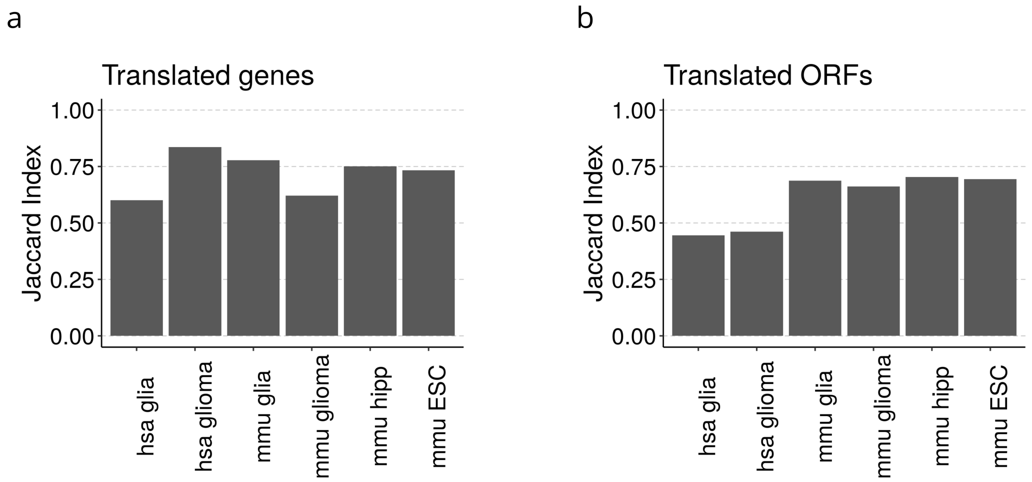

Supplementary Figure 7. (a) Overlap between the genes with at least one ORF predicted to be translated in ORQAS and in ORFquant calculated as a Jaccard Index (number of genes with at least one ORF predicted to be translated in the intersection of both methods divided by the total number with genes with at least one ORF predicted to be translated in any of the methods). **(b)** Overlap between ORF predicted to be translated in ORQAS and in ORFquant calculated as a Jaccard Index (number of ORFs predicted to be translated in the intersection of both methods divided by the total number ORFs predicted to be translated in any of the methods).

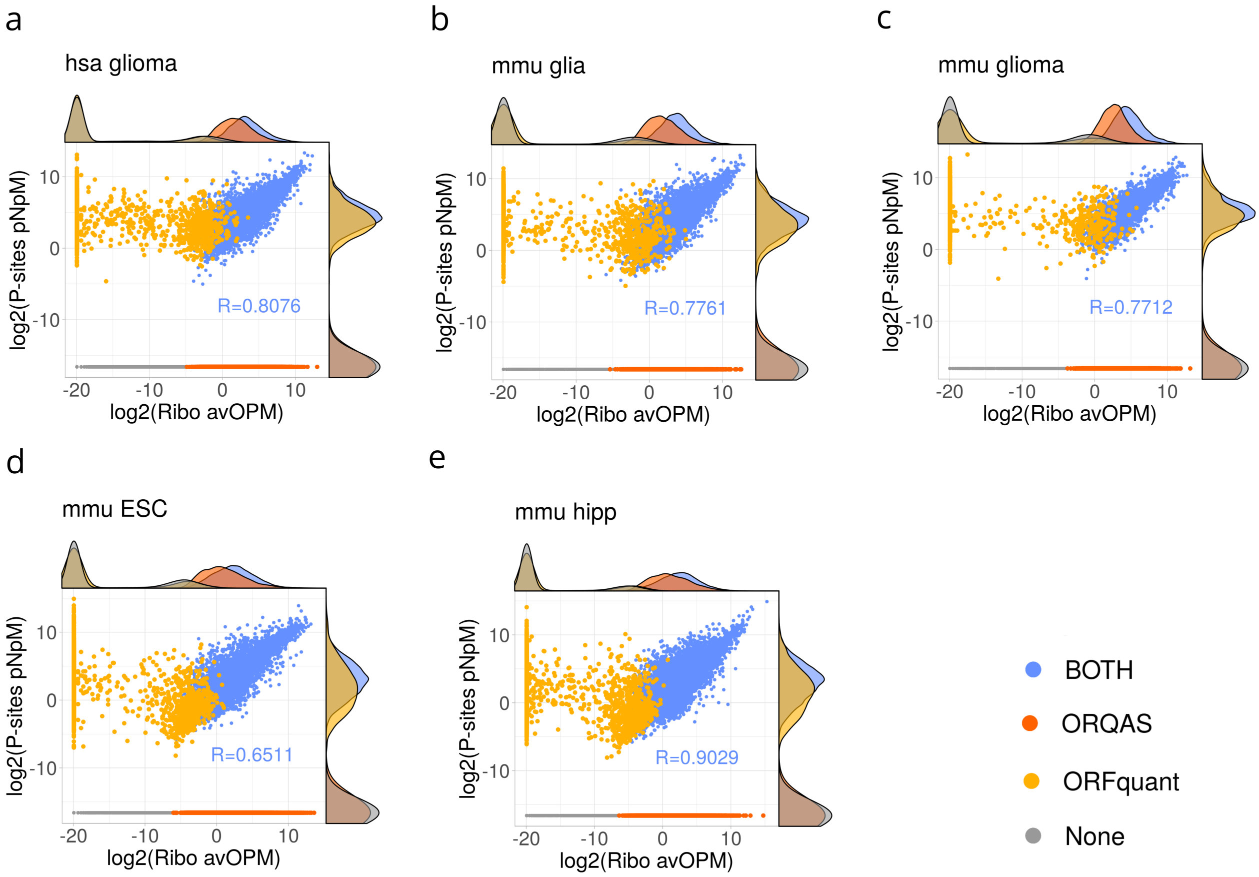

Supplementary Figure 8. Correlation between the average abundance in Ribosome space measured as ORFs per Million (OPM) by ORQAS and as P-sites per Nucleotide per Million (P-sites pNpM) by ORFquant for the samples of **(a)** human glioma (hsa glioma n=53420) and mouse samples of **(b)** glia (mmu glia n=37555), **(c)** glioma (mmu glioma n=37336), **(d)** Embryonic Stem Cells (mmu ESC n=37788) and **(e)** hippocampus (mmu hipp n=38039). Colours indicated if an ORF is found to be translated only by ORQAS (orange), only by ORFquant (yellow), by both methods (blue) or by none of them (grey).

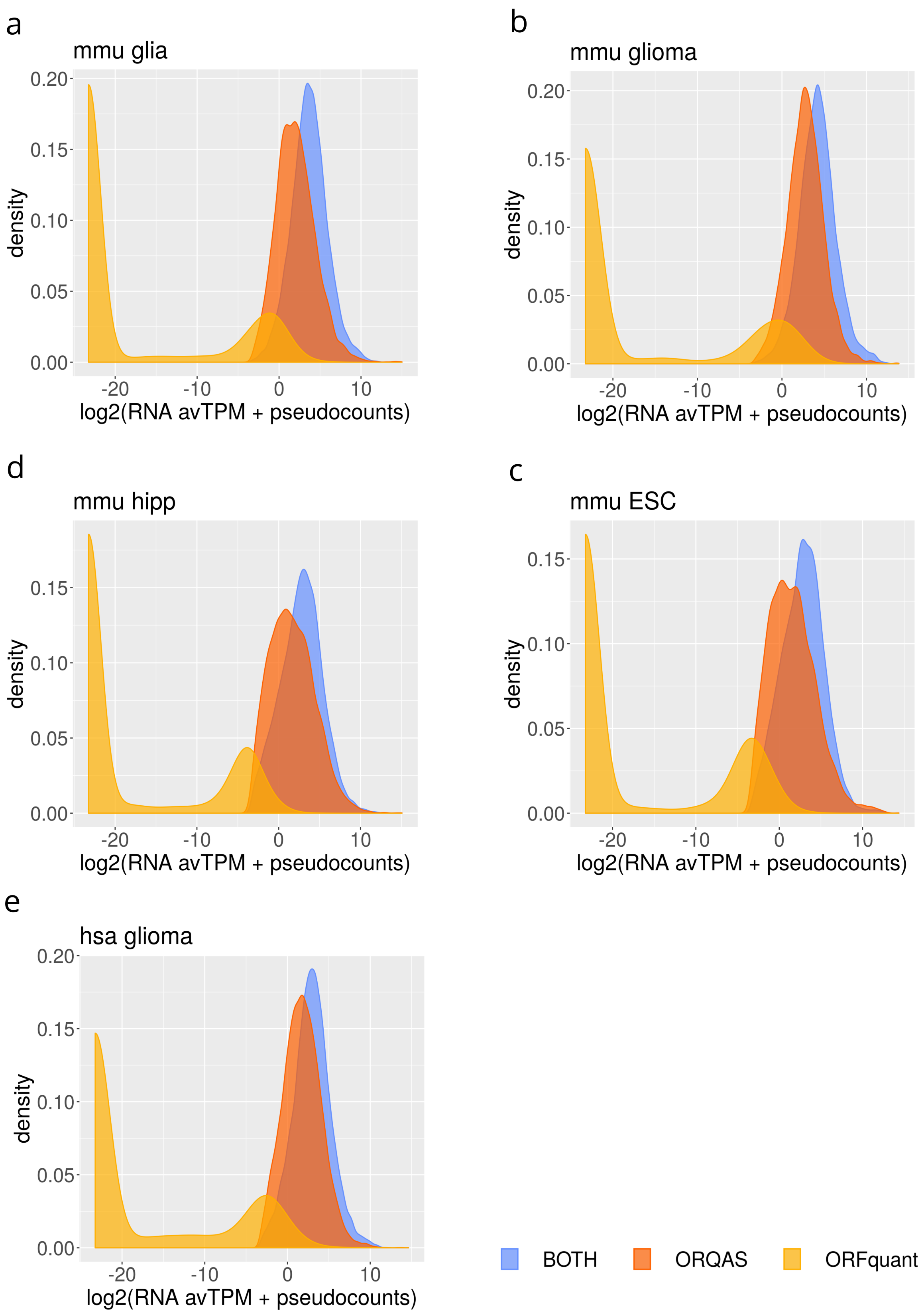

Supplementary Figure 9. Distribution of the average RNA expression measured in TPM for ORFs that are found to be translated only by ORQAS (orange), only by ORFquant (yellow) or by both methods (blue) in **(a)** mouse glia (mmu glia n=20925), **(b)** mouse glioma (mmu glioma n=17745), **(c)** mouse hippocampus (mmu hipp n=26093), **(d)** mouse Embryonic Stem Cells (mmu ESC n=22753) and **(e)** human glioma (hsa glioma n=29519).

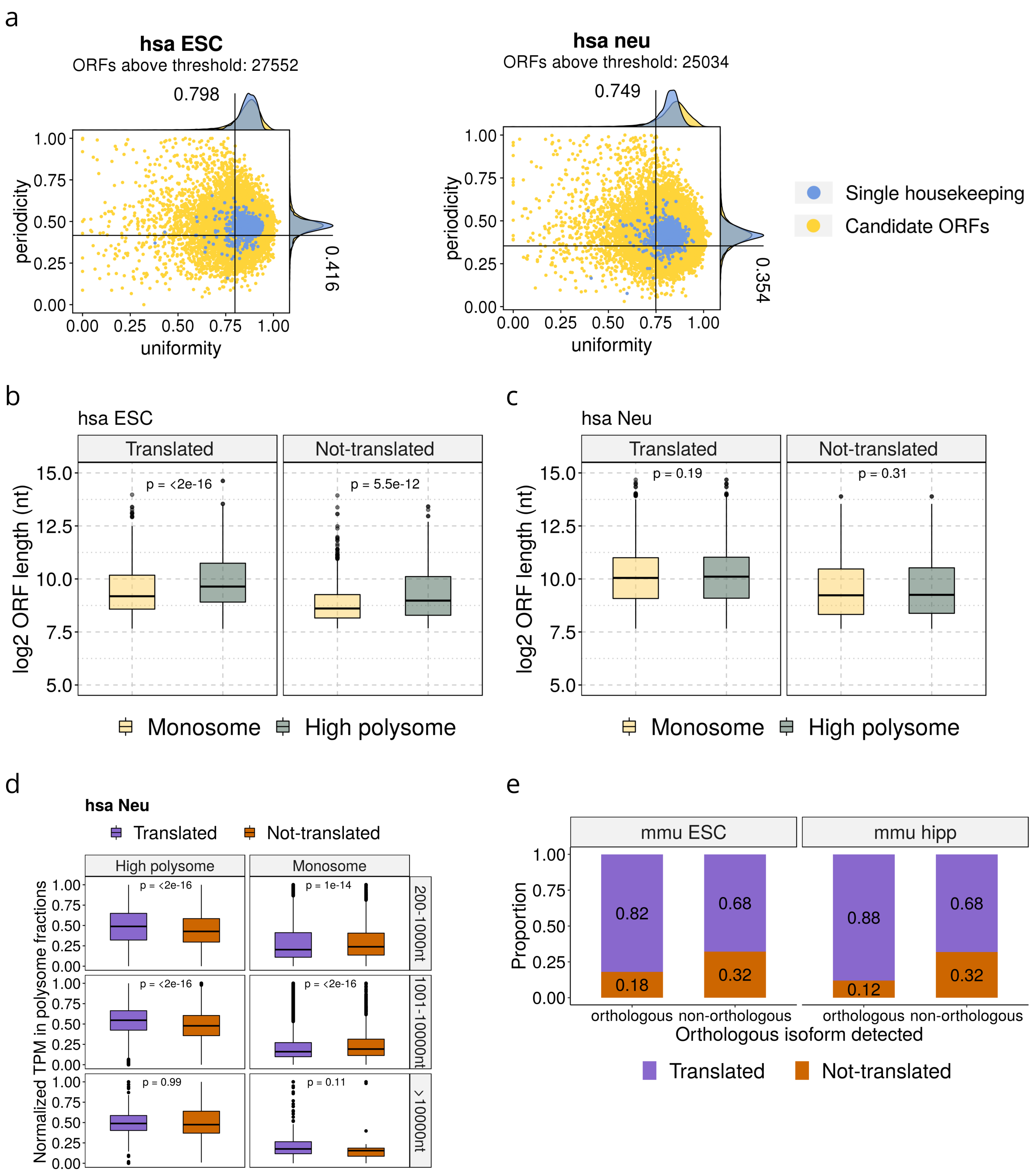

Supplementary Figure 10. (a) Uniformity (x axis) versus periodicity (y axis) for the ORFs with RNA expression TPM > 0.1 and at least 10 Ribo-seq reads assigned. In blue we indicate single-ORF genes with protein expression evidence in 37 tissues from THPA, and in yellow the rest of ORFs considered. Uniformity is measured as the percentage of maximum entropy and periodicity is measured in the first annotated frame. We show the data for the samples of human ESCs (left panel, n=37125) and human differentiated neurons (right panel, n=33358). **(b)** Distribution of the length in nucleotides of the ORFs expressed (normalized TPM > 0) only in the high polysome (left panels) and monosome (right panels) fractions of human Embryonic Stem Cells (hsa ESC) for translated isoforms (Wilcoxon test p-value 2.5e-05, high-polysome n=37425 and monosme n=31203) and for isoforms with RNA expression (TPM>0.1) but predicted as not translated (Wilcoxon test p-value

0.0463, high-polysome n=13279 and monosme n=11522). Box boundaries correspond to the first and the third quartiles, the median is indicated by a thick black line, top and bottom whiskers extend up to 1.5 times the interquartile range to the highest and smallest values, respectively, and outliers are indicated as black dots. **(c)** Distribution of the length in nucleotides of the ORFs expressed (normalized TPM > 0) only in the high polysome (left panels) and monosome (right panels) fractions of human Neural cells (hsa Neu) for translated isoforms (Wilcoxon test p-value 4.14e-15, high-polysome n=32496 and monosome n=28119) and for isoforms with RNA expression (TPM>0.1) but predicted as not translated (Wilcoxon test p-value 0.0028, high-polysome n=10680 and monosome n=9312). Box boundaries correspond to the first and the third quartiles, the median is indicated by a thick black line, top and bottom whiskers extend up to 1.5 times the interquartile range to the highest and smallest values, respectively, and outliers are indicated as black dots. **(d)** We show the distribution of the relative abundance in high polysome (left panels) and monosome (right panels) fractions of human Neural cells (hsa Neu) for translated isoforms and for isoforms with RNA expression (TPM>0.1) but predicted as not translated. The plot shows Wilcoxon test results for three different ORF lengths: 200-1000nt (high polysome Wilcoxon test p-value 6.35e-25, translated n=14555 and not-translated n=7235, and monosome Wilcoxon test p-value 1.05e-08, translated n=12733 and not-translated n=6330), 1001-10000nt (high polysome Wilcoxon test p-value 4.1e-53, translated n=17731 and not-translated n=2404, and monosome Wilcoxon test p-value 2.9e-10, translated n=15192 and not-translated n=2945) and longer than 10000nt (high polysome Wilcoxon test p-value 0.62, translated n=210 and not-translated n=41, and monosome Wilcoxon test p-vaue 0.53, translated n=194 and not-translated n=37). Box boundaries correspond to the first and the third quartiles, the median is indicated by a thick black line, top and bottom whiskers extend up to 1.5 times the interquartile range to the highest and smallest values, respectively, and outliers are indicated as black dots. **(e)** For the set of ORFs encoding a human-mouse orthologous protein pair (orthologous) and for those encoding proteins without an orthologous pair in mouse (non-orthologous) we plot the percentage that are predicted to be translated (translated) and the ones that did not pass the uniformity and periodicity thresholds (not-translated). We show here the results for mouse ESCs (Fisher's test p-value = 5.228e-124, orthologous n=12006 and non-orthologous n=9062) and for mouse hippocampus (Fisher's test p-value = 9.821e-311, orthologous n=13621 and non-orthologous n=10247).

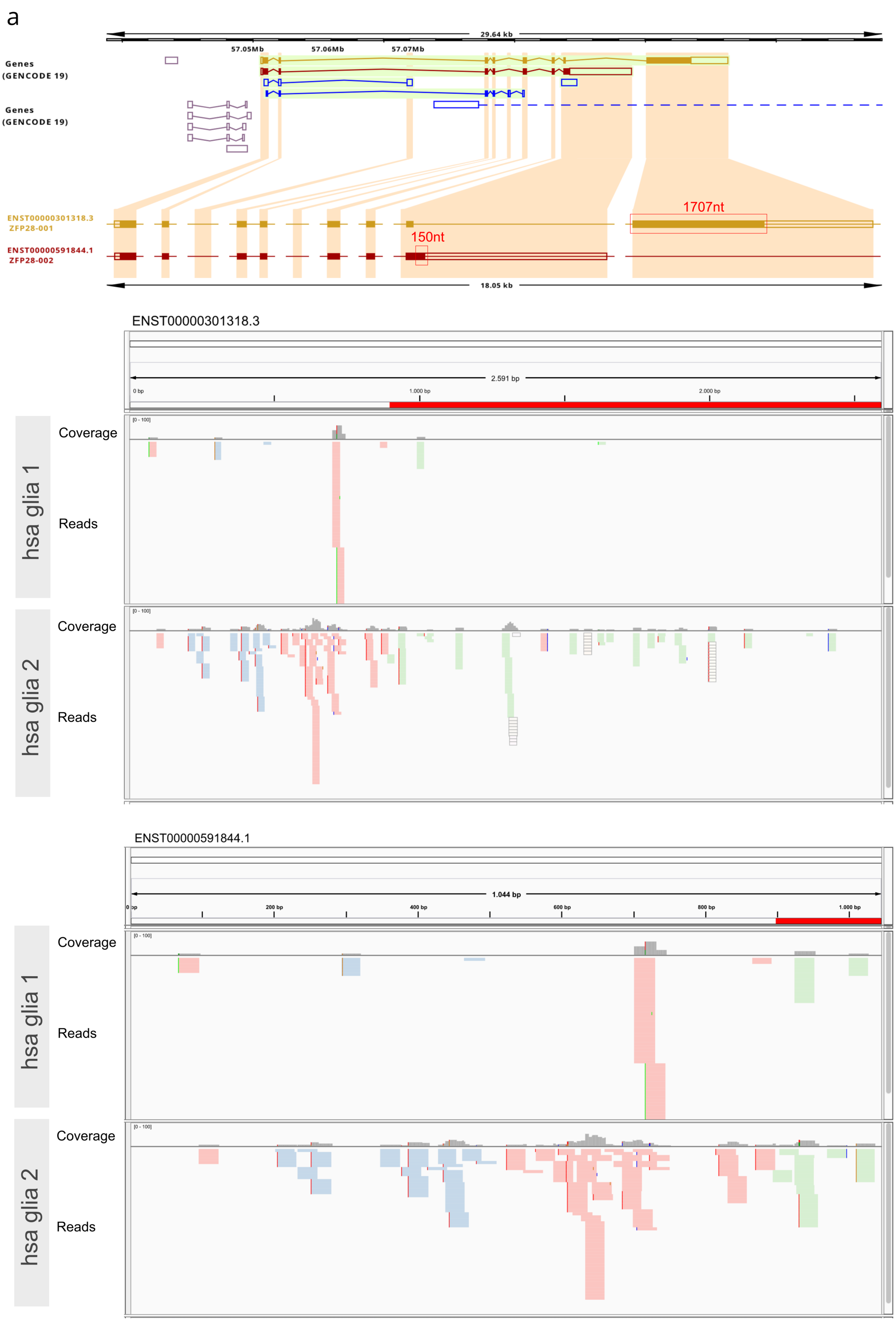

Supplementary Figure 11. (a) The upper panel shows the Ensembl annotation of the transcripts of *ZFP28* gene (ENSG00000196867) highlighting in red boxes the specific-sequence regions. Lower panels depict Ribo-seq reads in the human glia samples (hsa glia) mapping to the CDS of two isoforms of this gene ENST00000301318 (top) and ENST00000591844 (bottom) where specific-sequence regions are represented by the region highlighted in red. For each CDS we show the coverage of reads. The number of regions in the genome where the reads map is indicated in green (one), blue (two) and pink (three).

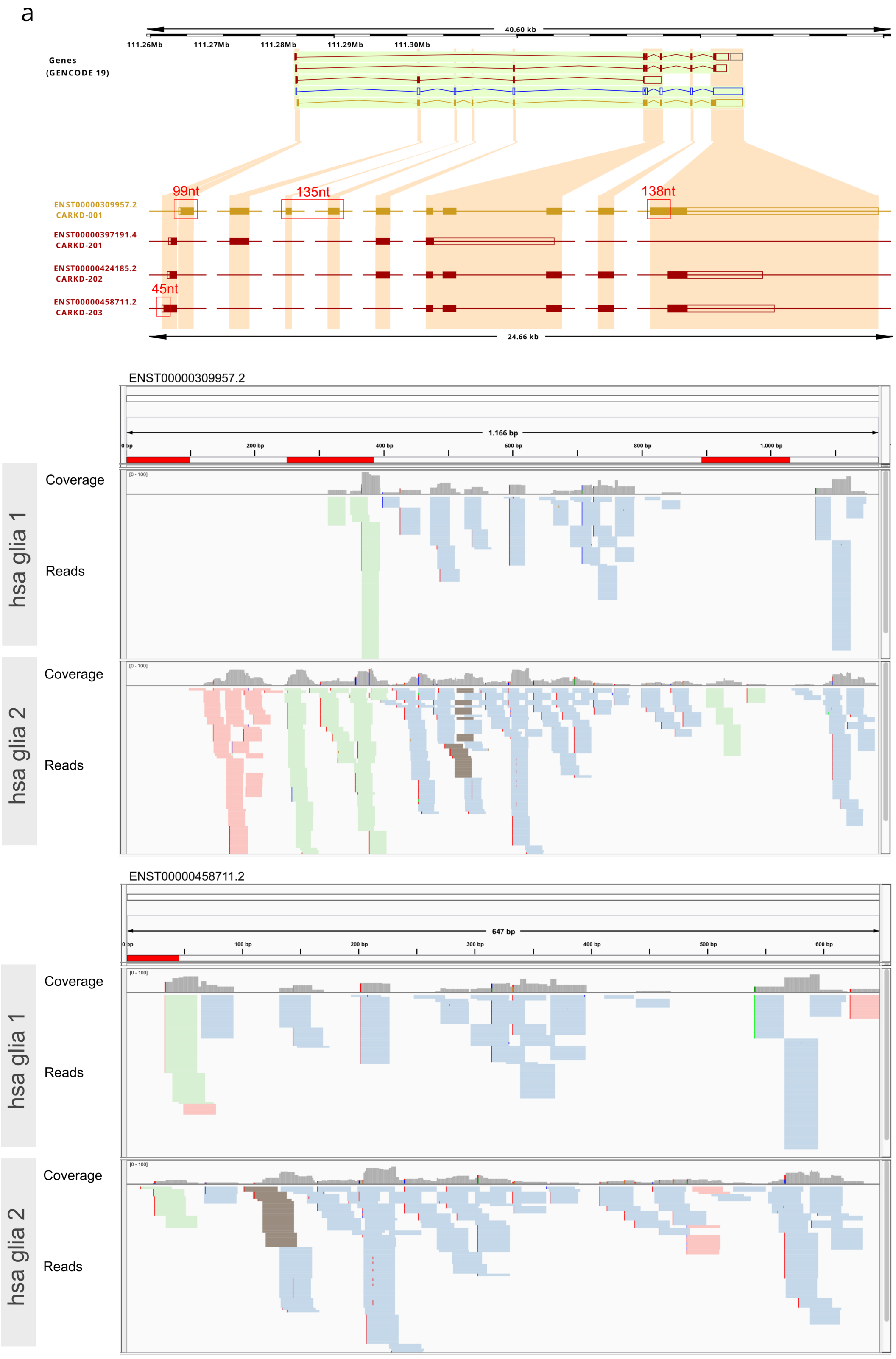

Supplementary Figure 12. (a) The upper panel shows the Ensembl annotation of the transcripts of *NAXD* gene (ENSG00000213995) highlighting in red boxes specific-sequence regions. Lower pannels depict Ribo-seq reads in the human glia samples (hsa glia) mapping to the CDS of two isoforms of this gene ENST00000309957 (top) and ENST00000458711 (bottom) where specific-sequence regions are represented by the region highlighted in red. For each CDS we show the coverage of reads. The number of regions in the genome where the reads map is indicated in green (one), blue (two), pink (three) and brown (four).

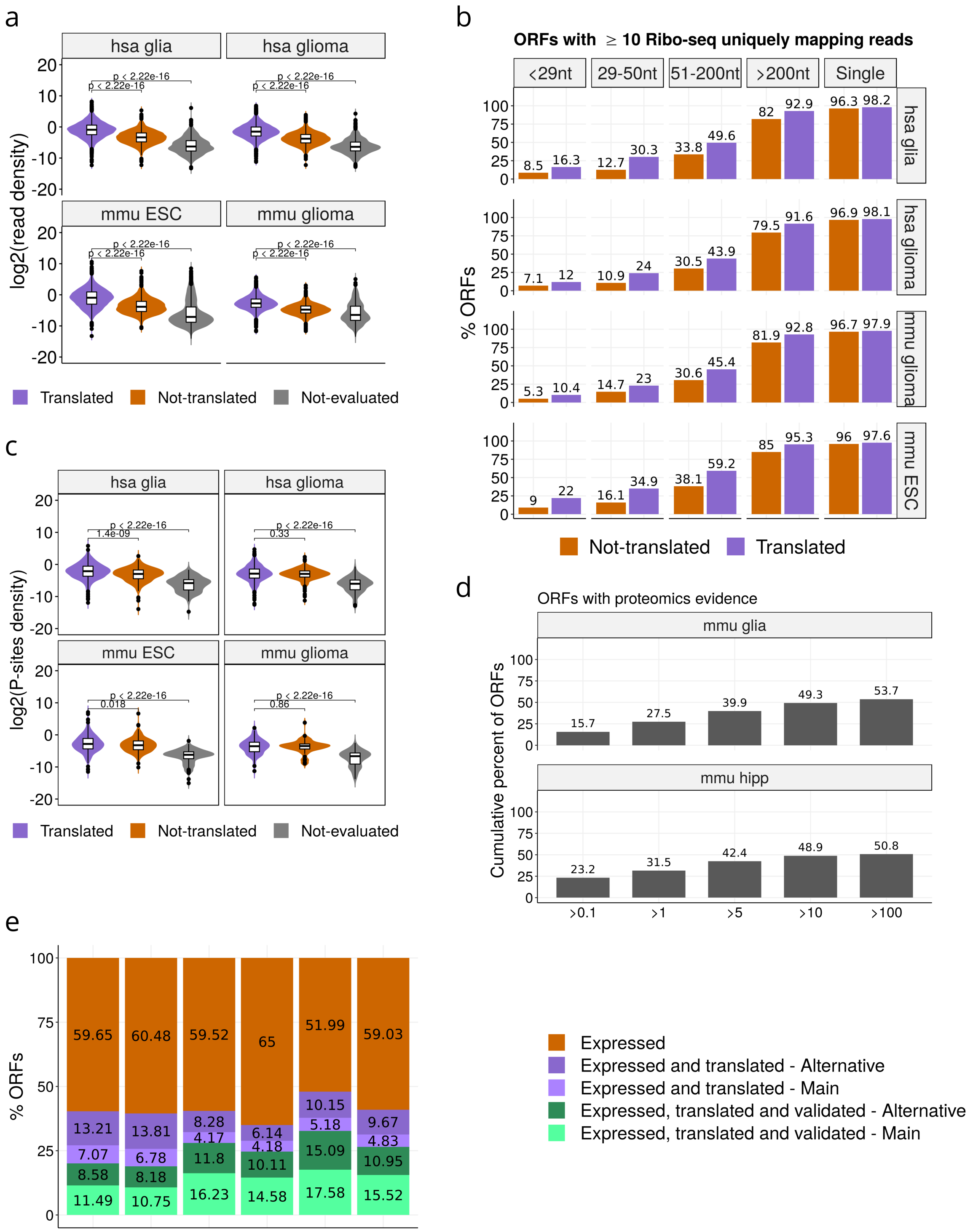

Supplementary Figure 13. (a) For the human samples of glia (hsa glia n=12417) and glioma (hsa glioma n=11033) and the mouse samples of glioma (mmu glioma n=12615) and Embryonic Stem Cells (mmu ESC n=16812) we show the density of Ribo-seq reads per nucleotide over the isoform-specific sequence regions, calculated as the uniquely mapping read-count over region length in log2 scale for isoform-specific sequences with at least 1 uniquely mapping read. Distributions are given for predicted translated isoforms, for isoforms that did not pass the threshold of uniformity and

periodicity (not translated), and for the isoforms with low expression (TPM<0.1) (not evaluated). Box boundaries correspond to the first and the third quartiles, the median is indicated by a thick black line, top and bottom whiskers extend up to 1.5 times the interquartile range to the highest and smallest values, respectively, and outliers are indicated as black dots. **(b)** For the human samples of glia (hsa glia) and glioma (hsa glioma) and the mouse samples of glioma (mmu glioma) and Embryonic Stem Cells (mmu ESC) the plot shows the percentage of regions with at least 10 uniquely mapping Ribo-seq reads in isoform-specific sequences over the total number of isoforms with an isoform-specific sequence defined according to the length of the region. **(c)** For the human samples of glia (hsa glia n=2410) and glioma (hsa glioma n=2513) and the mouse samples of glioma (mmu glioma n=1088) and Embryonic Stem Cells (mmu ESC n=1415) we show the density of Ribo-seq reads per nucleotide over the isoform-specific ORFs, calculated as the counts per nucleotide based on the estimated P-site positions over region length in log2 scale for isoform-specific ORFs with at least 1 P-site position count. Box boundaries correspond to the first and the third quartiles, the median is indicated by a thick black line, top and bottom whiskers extend up to 1.5 times the interquartile range to the highest and smallest values, respectively, and outliers are indicated as black dots. **(d)** For the mouse samples of glia (mmu glia) and hippocampus (mmu hipp), the plot shows the overall cumulative percentage of ORF-specific regions with 1 or more mass-spectrometry peptides according to increasing cut-offs of average RNA-seq abundance values (TPM). The plot shows the combined results for both types of regions: isoform-specific sequence regions and isoform-specific ORFs for isoforms predicted to be translated. **(e)** For each sample, and for all ORFs with sufficient RNA-seq expression (TPM > 0.1), we show the proportion of main isoforms and alternative isoforms predicted to be translated from Ribo-seq reads and the proportion that were validated. These plots do not include the genes with a single protein-coding isoform.

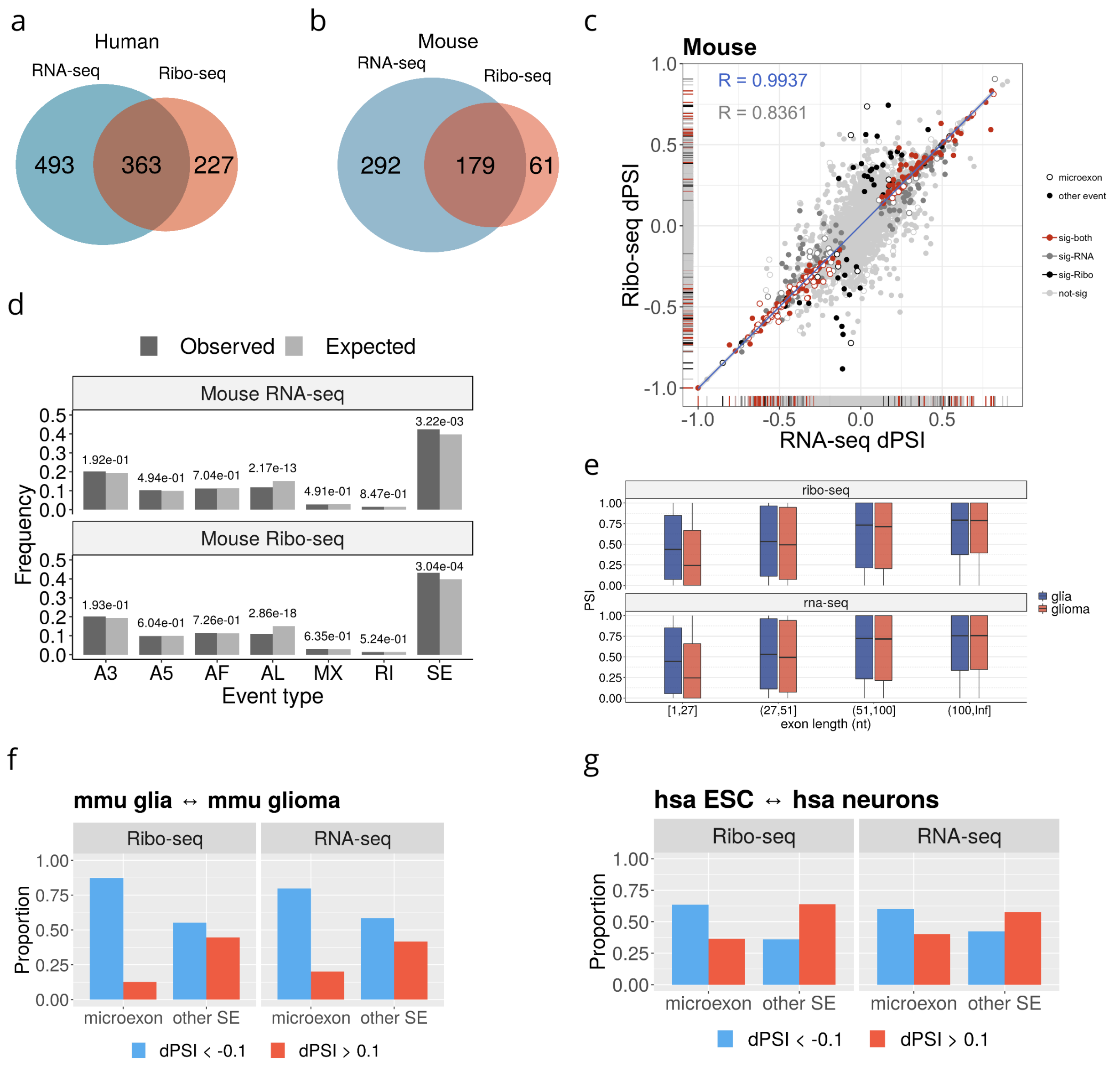

Supplementary Figure 14. (a) Overlap of events changing significantly (SUPPA dPSI > 0.1 and p-value < 0.05) with RNA-seq and with Ribo-seq for human. **(b)** Overlap of events changing significantly (SUPPA dPSI > 0.1 and p-value < 0.05) with RNA-seq and with Ribo-seq for mouse. **(c)** Correlation of changes in splicing and translation in events in mouse. **(d)** Proportions of events calculated in RNA space (upper panel) or Ribosome space (lower panel). In light grey we show the proportion of alternative splicing events calculated with SUPPA that overlap coding regions in mouse, whereas in dark grey we show the events that show a significant change using RNA-seq comparing mouse glia and glioma. Even types are alternative 3'ss (A3) and 5'ss (A5), alternative first (AF) and last (AL) exon, mutually exclusive (MX) exon, retained introns (RI) and skipping exon (SE). There is significant enrichment of SE events for RNA (Fisher's test p-value = 3.22e-03) and Ribo-seq (Fisher's test p-value = 3.04e-04); and significant depletion of AL events for RNA (Fisher's test p-value 2.17e-13) and Ribo-seq (Fisher's test p-value 2.86e-18). **(e)** Inclusion level in PSI values of skipping exon (SE) events according to the length of the central exon (in nucleotides) for the

human glia and glioma samples. Exon lengths in <52 nucleotides are defined as microexons. **(f)** Enrichment of microexons with an impact in RNA splicing and ORF translation in mouse from the comparison of glia and glioma samples. In the figure, dPSI is used to indicate the difference in relative abundance in both RNA and Ribosome spaces. **(g)** Difference in high polysome fraction, measured as dPSI, between neuronal samples and ESCs (y axis) for microexons with a significant change in Ribosome space. As before, dPSI indicates the difference in relative abundance in Ribosome space.

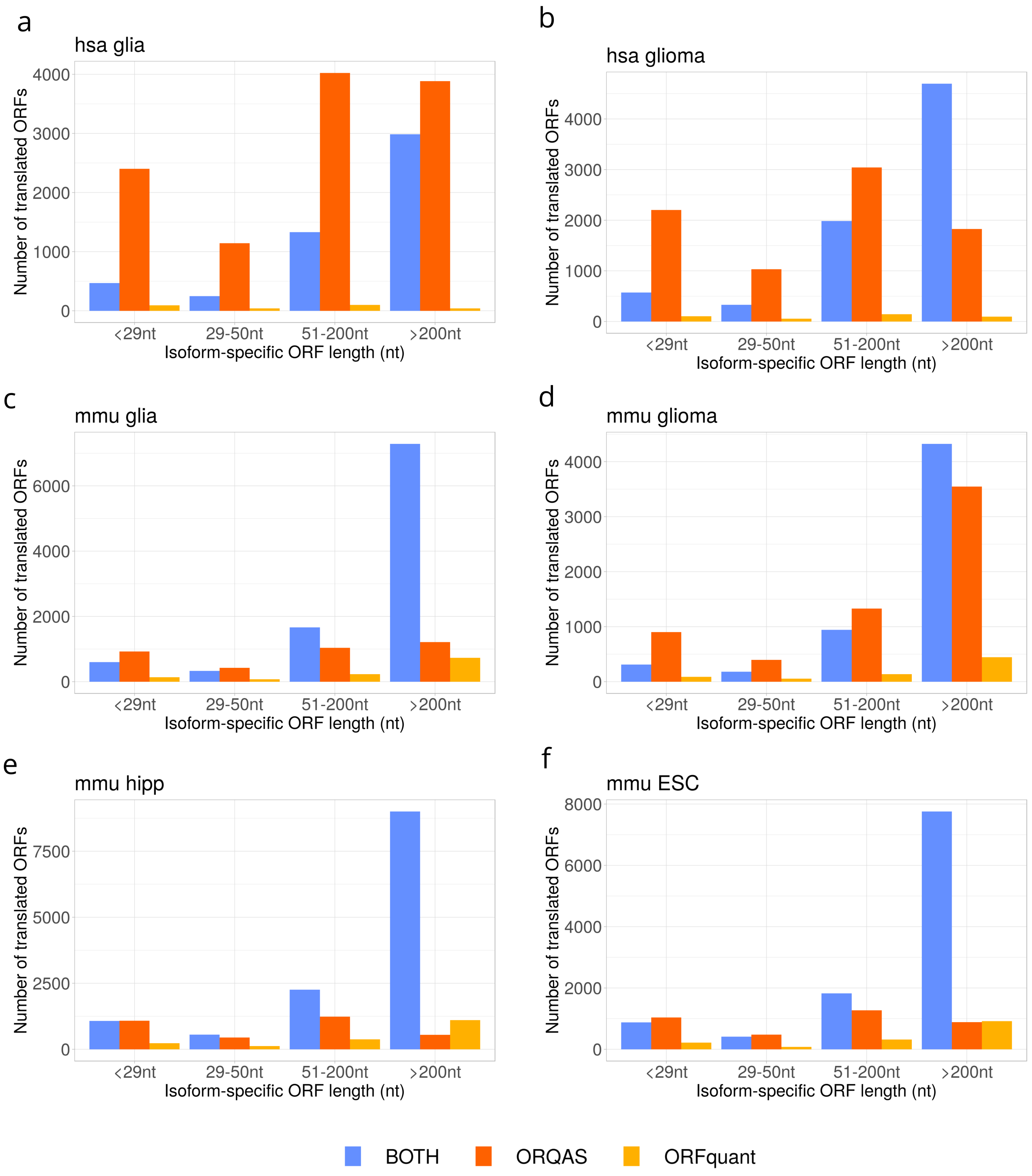

Supplementary Figure 15. Number of ORFs containing isoform-specific ORF regions that are found to be translated only by ORQAS (orange), only by ORFquant (yellow) or by both methods (blue) according to the isoform-specific ORF length for the samples of **(a)** human glia (hsa glia), **(b)** human glioma (hsa glioma), **(c)** mouse glia (mmu glia), **(d)** mouse glioma (mmu glioma), **(e)** mouse hippocampus (mmu hipp) and **(f)** mouse Embryonic Stem Cells (mmu ESC).

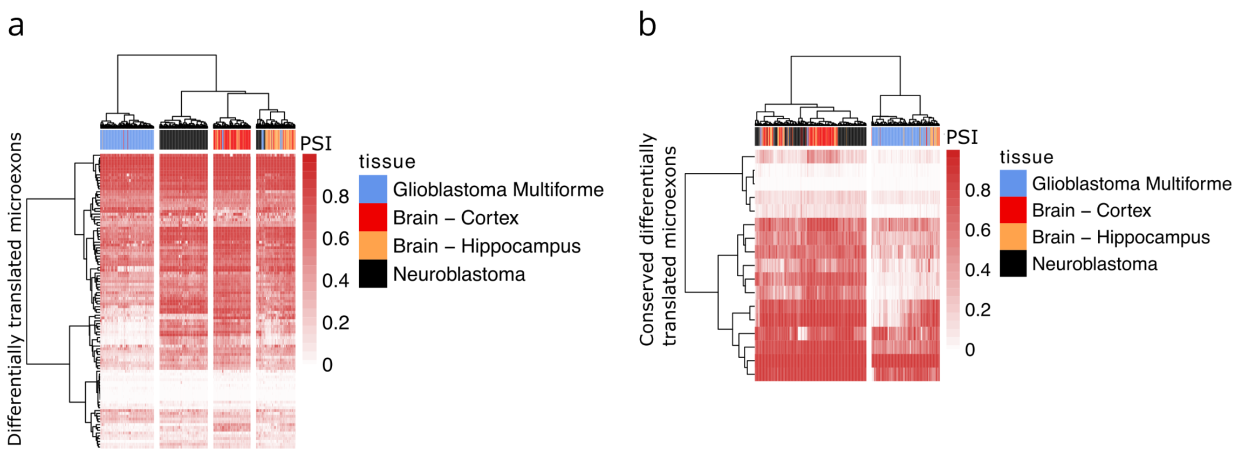

Supplementary Figure 16. (a) Patterns of inclusion of differentially translated microexons in normal brain and hippocampus samples from GTEX, glioblastoma multiforme from TCGA and neuroblastoma from TARGET. The heatmap shows the Percent Spliced In (PSI) values for each sample. **(b)** Patterns of inclusion of conserved differentially translated microexons in normal brain and hippocampus samples from GTEX, glioblastoma multiforme (GBM) from TCGA and neuroblastoma (NB) from TARGET. The heatmap shows the Percent Spliced In (PSI) values for each sample.

Dataset	Species	Sample number	Geo accession	Reference
**Hsa glia**	Homo sapiens	2	GSE51424	Gonzalez et al 2014
**Hsa glioma**	Homo sapiens	2	GSE51425	Gonzalez et al 2014
**Mmu glia**	Mus musculus	3	GSE51426	Gonzalez et al 2014
**Mmu glioma**	Mus musculus	3	GSE51427	Gonzalez et al 2014
**Mmu hipp**	Mus musculus	3	GSE72064	Cho et al 2015
**Mmu ESC**	Mus musculus	2	GSE89011	Sugiyama et al 2017

Supplementary Table 1. Information of the samples used in this study.

Dataset	Total ORFs	Expressed (TPM >0.1)	Evidence of translation		Validation				Overall validation		
			Count	% of the expressed	Reads in specific sequence regions	P-sites in specific ORF regions	Peptides in specific ORF regions	Isoform conservation	Count	% of the expressed	% of the translated
**Hsa glia**	84024	48967	20785	42,45	6301	6532	NA	9151	11564	23,62	55,64
**Hsa glioma**	84024	50010	20709	41,41	5723	5892	NA	9014	11138	22,27	53,78
**Mmu glia**	48924	31298	14777	47,21	7220	7500	6006	9569	11714	37,43	79,27
**Mmu glioma**	48924	31080	13019	41,89	6432	6450	NA	8724	10473	33,70	80,44
**Mmu hipp**	48924	31850	17515	54,99	9486	9490	6894	10529	13715	43,06	78,30
**Mmu ESC**	48924	30227	14794	48,94	7736	7783	NA	8649	11299	37,38	76,38

Supplementary Table 2. Number of expressed (RNA) ORFs, number of predicted translating ORFs, number of cases validated by each method, and total number with any of the validations (and percentages).

Dataset	Lowly expressed ORFs (TPM <= 0.1)	Evidence of translation	Evidence of translation (%)
**Hsa glia**	18356	177	0,96
**Hsa glioma**	17724	126	0,71
**Mmu glia**	12415	18	0,14
**Mmu glioma**	12190	13	0,11
**Mmu hipp**	12052	206	1,71
**Mmu ESC**	14415	85	0,59

Supplementary Table 3. Table with the ORFs with evidence of translation (false positives) among the ones with low RNA expression (average TPM <= 0.1).

Dataset	Expressed (TPM > 0.1)*	Translated: Main isoform	Translated: Alternative isoform	Validated: Main isoform	Validated: Alternative isoform
**Hsa glia**	45034	8357	9814	5174	3866
**Hsa glioma**	46100	8080	10140	4956	3772
**Mmu glia**	24154	4928	4849	3921	2850
**Mmu glioma**	23963	4495	3893	3493	2422
**Mmu hipp**	24339	5540	6144	4280	3673
**Mmu ESC**	23627	4809	4872	3667	2588

SupplementaryTable 4. Number of expressed (RNA) ORFs, number of predicted translating ORFs, number of cases validated by each method, and total number with any of the validations for the isoforms that are not singletons (Main and Alternative isoforms). *Without singletons
