# Supplementary material for: Ribosome profiling at isoform level reveals evolutionary conserved impacts of differential splicing on the proteome
Source: Nat Commun. 2020 Apr 14;11:1768. doi: 10.1038/s41467-020-15634-w (PMC7156646; doi:10.1038/s41467-020-15634-w)
Supplement: Supplementary file 3 — Reporting Summary [file 41467_2020_15634_MOESM3_ESM.pdf]

nature research

Corresponding author(s): *Double-blind peer review submissions: write DBPR and your manuscript number here instead of author names.*

Last updated by author(s): May 3, 2019

Reporting Summary

Nature Research wishes to improve the reproducibility of the work that we publish. This form provides structure for consistency and transparency in reporting. For further information on Nature Research policies, see Authors & Referees and the Editorial Policy Checklist.

Statistics

For all statistical analyses, confirm that the following items are present in the figure legend, table legend, main text, or Methods section.

n/a	Confirmed	
☐	☒	The exact sample size (*n*) for each experimental group/condition, given as a discrete number and unit of measurement
☐	☒	A statement on whether measurements were taken from distinct samples or whether the same sample was measured repeatedly
☐	☒	The statistical test(s) used AND whether they are one- or two-sided<br>*Only common tests should be described solely by name; describe more complex techniques in the Methods section.*
☐	☒	A description of all covariates tested
☐	☒	A description of any assumptions or corrections, such as tests of normality and adjustment for multiple comparisons
☐	☒	A full description of the statistical parameters including central tendency (e.g. means) or other basic estimates (e.g. regression coefficient) AND variation (e.g. standard deviation) or associated estimates of uncertainty (e.g. confidence intervals)
☐	☒	For null hypothesis testing, the test statistic (e.g. *F*, *t*, *r*) with confidence intervals, effect sizes, degrees of freedom and *P* value noted<br>*Give P values as exact values whenever suitable.*
☒	☐	For Bayesian analysis, information on the choice of priors and Markov chain Monte Carlo settings
☒	☐	For hierarchical and complex designs, identification of the appropriate level for tests and full reporting of outcomes
☒	☐	Estimates of effect sizes (e.g. Cohen's *d*, Pearson's *r*), indicating how they were calculated

Our web collection on statistics for biologists contains articles on many of the points above.

Software and code

Policy information about availability of computer code

Data collection	Data was downloaded manually from public repositories using specific accessions (listed below). We did not used any specific software.
Data analysis	All software used to analyse the datasets is described in https://github.com/comprna/ORQAS/<br>Custom software specifically developed for this publication (ORQAS pipeline) is available from the same website and is implemented in Python 3.5. Additionally, we have used SUPPA (https://github.com/comprna/SUPPA), Salmon (https://github.com/COMBINE-lab/salmon), STAR (https://github.com/alexdobin/STAR), Ribomap (http://www.cs.cmu.edu/~ckingsf/software/ribomap/)

For manuscripts utilizing custom algorithms or software that are central to the research but not yet described in published literature, software must be made available to editors/reviewers. We strongly encourage code deposition in a community repository (e.g. GitHub). See the Nature Research guidelines for submitting code & software for further information.

Data

Policy information about availability of data

All manuscripts must include a data availability statement. This statement should provide the following information, where applicable:

- Accession codes, unique identifiers, or web links for publicly available datasets
- A list of figures that have associated raw data
- A description of any restrictions on data availability

Our analysis used data from GEO (https://www.ncbi.nlm.nih.gov/geo/) with accessions: GSE51424, GSE72064, GSE89011, and GSE100007; data from PRIDE (https://www.ebi.ac.uk/pride/archive/) with accessions PRD000010, PXD000349, PXD001786, PRD000363, PXD000311, PXD001135 and PRD000522;
From THPA we downloaded protein expression from the normal samples from (https://www.proteinatlas.org/download/normal_tissue.tsv.zip);
TARGET, TCGA and GTEX datasets used (transcript abundances) were downloaded from the UCSC XENA browser Xenabrowser de UCSC (https://xenabrowser.net/datapages/?dataset=TcgaTargetGtex_rsem_isoform_tpm - TCGA TARGET GTEx dataset).

List of figures that show the associated raw data: Figures 1(b,c,d,e), 2(a,c,d,e), 3(b,c,d,e,f), 4(b,c,d,e,f,g), 5(b,c,d,e)

There are no restrictions on data availability.

Field-specific reporting

Please select the one below that is the best fit for your research. If you are not sure, read the appropriate sections before making your selection.

☒ Life sciences ☐ Behavioural & social sciences ☐ Ecological, evolutionary & environmental sciences

For a reference copy of the document with all sections, see nature.com/documents/nr-reporting-summary-flat.pdf

Life sciences study design

All studies must disclose on these points even when the disclosure is negative.

Sample size	We used all data available in the public repositories indicated above. The only limitation was that we required a minimum of 2 biological replicates in order to calculate differential splicing between conditions with SUPPA.
Data exclusions	No data was excluded for the analysis.
Replication	We did not perform any experimental procedures. All data was downloaded from public repositories. Further information about the experimental procedures involved in the generation of the data in the original publications can be found through the accessions provided above. The experimental data used had 2 replicates per condition, and both were used in all analyses.
Randomization	We did not perform any experimental procedures. All data was downloaded from public repositories. Further information about the experimental procedures involved in the generation of the data in the original publications can be found through the accessions provided above.
Blinding	We did not perform any experimental procedures. All data was downloaded from public repositories. Further information about the experimental procedures involved in the generation of the data in the original publications can be found through the accessions provided above.

Reporting for specific materials, systems and methods

We require information from authors about some types of materials, experimental systems and methods used in many studies. Here, indicate whether each material, system or method listed is relevant to your study. If you are not sure if a list item applies to your research, read the appropriate section before selecting a response.

Materials & experimental systems

n/a	Involved in the study	
☒	☐	Antibodies
☒	☐	Eukaryotic cell lines
☒	☐	Palaeontology
☒	☐	Animals and other organisms
☒	☐	Human research participants
☒	☐	Clinical data

Methods

n/a	Involved in the study	
☒	☐	ChIP-seq
☒	☐	Flow cytometry
☒	☐	MRI-based neuroimaging
