# Supplementary material for: Ribosome profiling at isoform level reveals evolutionary conserved impacts of differential splicing on the proteome
Source: Nat Commun. 2020 Apr 14;11:1768. doi: 10.1038/s41467-020-15634-w (PMC7156646; doi:10.1038/s41467-020-15634-w)
Supplement: Supplementary file 4 — Description of Additional Supplementary Files [file 41467_2020_15634_MOESM4_ESM.pdf]

Description of Additional Supplementary Files

File Name: Supplementary Data 1
Description: Translational state of the isoforms in the analyzed datasets. A tab separated file including the following fields: species and condition of the sample (dataset), ORF ID indicated by all the Ensembl transcript IDs that encode the same ORF concatenated with a semicolon (ORF), gene ID, average RNA expression from all replicates (RNA_avTPM), the average ORF expression from all replicates (Ribo_avOPM), number of Ribo-seq reads from all replicates collapsed (ribo_reads), periodicity in the coding frame (periodicity_f1), uniformity as a Percentage of Maximum Entropy (uniformity_pme), translation status assigned after running ORQAS pipeline (translated): translated, nottranslated or not-evaluated, whether the isoform corresponds to the control set of housekeeping singleisoform genes with Mass Spectrometry evidence of expression (category), whether the ORF is encoded by (category2) a single-ORF gene - Single, the most abundant isoform according to RNA-seq - Main isoform, the second most abundant - Secondary isoform, or by any of the remaining isoforms - Other isoforms, starts of the isoform specific sequence regions in the ORF coordinates (starts_urseq), ends of the isoform specific sequence regions in the ORF coordinates (ends_urseq), total length of the isoform specific sequence regions (len_urseq), number of uniquely mapping reads in the isoform specific sequence regions (reads_urseq), uniquely mapping read density in the isoform specific sequence regions (density_urseq), starts of the isoform specific ORF regions in the ORF coordinates (starts_urorf), ends of the isoform specific ORF regions in the ORF coordinates (ends_urorf), total length of the isoform specific ORF regions (len_urorf), number of P-sites in the isoform specific ORF regions (counts_urorf), P-site density in the isoform specific ORF regions (density_urseq), number of peptides from PRIDE database mapping to isoform specific ORF regions (n_pep).

File Name: Supplementary Data 2
Description: Splicing events in human glia and glioma samples. A tab separated file including the following fields: event coordinates (event_id), PSI values for each sample, whether the event is an alternative 3’ss/5’ss - A3/A5, alternative first/last exon - AF/AL , mutually exclusive exon - MX, retained intron - RI and skipping exon – SE (type), whether an SE event is categorized as a microexon (microexon), coordinates of predicted orthologous SE events in mouse (orthologous_SE), Ensembl gene ID (gene_id), Ensembl transcript IDs for the transcripts involved in the event (alternative_transcripts) and Ensembl transcript IDs of all transcripts in the gene (total_transcripts).

File Name: Supplementary Data 3
Description: Splicing events in human ESC and neural samples. A tab separated file including the following fields: event coordinates (event_id), PSI values for each sample, whether the event is an alternative 3’ss/5’ss - A3/A5, alternative first/last exon - AF/AL , mutually exclusive exon - MX, retained intron - RI and skipping exon – SE (type), whether an SE event is categorized as a microexon (microexon), coordinates of predicted orthologous SE events in mouse (orthologous_SE), Ensembl gene ID (gene_id), Ensembl transcript IDs for the transcripts involved in the event (alternative_transcripts) and Ensembl transcript IDs of all transcripts in the gene (total_transcripts).

File Name: Supplementary Data 4
Description: Splicing events in mouse glia and glioma samples. A tab separated file including the following fields: event coordinates (event_id), PSI values for each sample, whether the event is an alternative 3’ss/5’ss - A3/A5, alternative first/last exon - AF/AL , mutually exclusive exon - MX, retained intron - RI and skipping exon – SE (type), whether an SE event is categorized as a microexon (microexon), coordinates of predicted orthologous SE events in human (orthologous_SE), Ensembl gene ID (gene_id), Ensembl transcript IDs for the transcripts involved in the event (alternative_transcripts) and Ensembl transcript IDs of all transcripts in the gene (total_transcripts).
